# Embedded Adaptive Optics for Ubiquitous Lab-on-a-Chip Readout on Intact Cell Phones

**DOI:** 10.3390/s120708586

**Published:** 2012-06-26

**Authors:** Pakorn Preechaburana, Anke Suska, Daniel Filippini

**Affiliations:** 1 Optical Devices Laboratory, Division of Applied Physics, IFM–Linköping University, SE-58183 Linköping, Sweden; E-Mails: anksu@ifm.liu.se (A.S.); danfi@ifm.liu.se (D.F.); 2 Department of Physics, Faculty of Science and Technology, Thammasat University, Pathum Thani 12121, Thailand

**Keywords:** adaptive optics, lab-on-a-chip readout, optical chemical sensing, ubiquitous sensing, cell phones

## Abstract

The evaluation of disposable lab-on-a-chip (LOC) devices on cell phones is an attractive alternative to migrate the analytical strength of LOC solutions to decentralized sensing applications. Imaging the micrometric detection areas of LOCs in contact with intact phone cameras is central to provide such capability. This work demonstrates a disposable and morphing liquid lens concept that can be integrated in LOC devices and refocuses micrometric features in the range necessary for LOC evaluation using diverse cell phone cameras. During natural evaporation, the lens focus varies adapting to different type of cameras. Standard software in the phone commands a time-lapse acquisition for best focal selection that is sufficient to capture and resolve, under ambient illumination, 50 μm features in regions larger than 500 × 500 μm^2^. In this way, the present concept introduces a generic solution compatible with the use of diverse and unmodified cell phone cameras to evaluate disposable LOC devices.

## 1. Introduction

Disposable lab-on-a-chip (LOC) devices are an attractive platform for the implementation of compact and robust analytical tests, which minimize sample volumes and simplify the handling of the measurements, both important factors in distributed analyses [1,2]. Among the diverse possibilities existing for LOC readout, optical methods [3] are those relevant to this work.

Disposable LOC devices have been demonstrated for numerous sensing and clinical applications [3], however, their dissemination is restricted by the instrumentation required for readout. LOC solutions for point of care (POC) or other distributed detections [2] are typically associated with dedicated and specific *off-chip* readers [4,5].

Thus, although LOC devices can be disposable and deployable at a large scale, the availability of readers and their specific characteristics restrict the dissemination of analyses based on this technology. Therefore, if disposable LOC devices could be evaluated using generic and common platforms, such as cell phones, the benefits of this technology could be made ubiquitous.

On the other hand, dedicated instruments for chemical sensing, both compact or sizable, which make use of regular cell phones for imaging [6,7] and communication purposes [8] have been demonstrated in recent years. In some cases the cell phones are embedded within the instrument [8] and permanently modified, whereas in other examples there is only a temporary connection [6] to the instrument and the phone remains usable for its natural purpose. In both cases, the components additional to the phone are not common and restrict the ubiquity of the combined solution.

In contrasts, ubiquitous chemical sensing approaches have been developed during the past ten years to take advantage of mass-produced consumer electronic devices such as flatbed scanners [9], DVD/CD drives [10], computer sets [11], and also cell phones [12]. In these examples, components are sensibly combined to minimize additional interfacing elements that could restrict ubiquity. In this work we follow these principles aiming at solution that can be integrated in disposable LOC devices for ubiquitous sensing.

Here we investigate *off-chip* readout of disposable LOC devices on cell phones without additional accessories and using adaptive optics integrated in the same disposable LOC that will be evaluated. The device sits on the camera surface, which provides a standard mechanical support for the device, optical coupling and a compact configuration. The device temporarily sticks on the camera during evaluation and is disposed afterwards.

Intact cell phone cameras cannot focus at the short distances required by this concept, and the LOC must incorporate a refocusing element to image its micrometric detection area. Simple fixed lenses can be implemented for a particular camera type [13]; however, different brands and models have slightly different optical designs, and a generic solution to this problem demands to adapt to all of these conditions with a unified concept. Adaptive optics is central for autofocusing and can be implemented in different ways [14–17] as dedicated components, but in this work we seek a solution that can be embedded in disposable LOC devices, such as a sessile drop [18–20] complemented by data analysis.

Here we demonstrate a disposable morphing lens concept that can be integrated in the LOC device, and operates on different phone and computer cameras, rendering these platforms capable to image the micrometric detection regions necessary for the evaluation of LOC devices.

## 2. Experimental Section

### 2.1. Lens Supporting Device

The lens supporting part of the LOC device was made from Dow Corning Sylgard 184 PDMS (polydimethylsiloxane [21]) with a base/curing agent ratio of 10:1, as negative replicas of a SU-8 (10) template (Microchem Corp., Newton, MA, USA) created with a refined micro projection lithography system (MPLS) described elsewhere [22]. The template created a 30 μm deep circular depression that confines the liquid lens.

**Figure 1.**
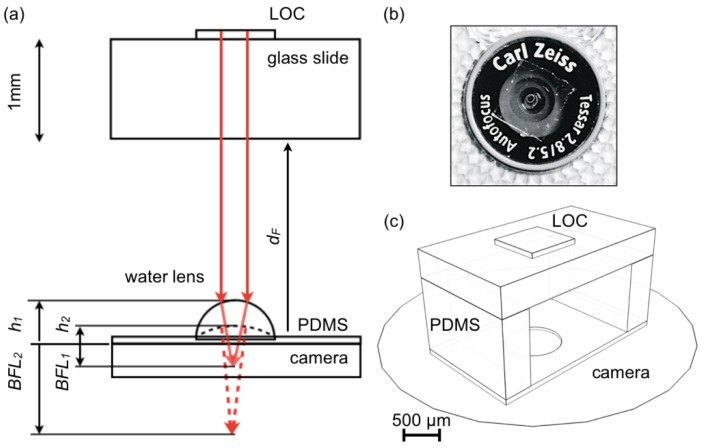
(**a**) Cross-section of the measuring device. The liquid lens is represented at two different volumes with the associated back focal lengths (*BFL*). The forward distance (*d_F_*) is 1 and 2 mm in the experiments and it is defined by PDMS structural elements; (**b**) Nokia 6720 cell phone rear camera with a PDMS lens substrate adhered to its surface; (**c**) 3D scheme of the device including the integrated adaptive focusing element.

To fabricate the PDMS substrate, 10 g of Dow Corning Sylgard 184 base and 1 g of curing agent were mixed and stirred in a cup for 5 min. The mixture was degased in a desiccator connected with a rotary pump for 45 min, and afterwards poured on a SU-8(10) template. The PDMS film was then cured at 65 °C for 2 h in an environmental oven. The result was an adhesive microstructured 150 μm thick film that was cut with a blade and placed on phone cameras, serving as support of the liquid lens, and the PDMS elements that hold the LOC substrate (Figure 1(c)).

### 2.2. Test Structures

The LOCs devices used for testing are 3D chambers created using a refined MPLS approach [22]. In order to make closed chambers with their own roof, the exposure depth of a SU-8/S1818 mixture is precisely controlled, as described in a previous work [22]. The mixture of SU-8 (50) (Microchem Corp., Newton, MA, USA) with 10% in volume of S1818 G2 (Rohm and Hass) enhances light absorption for the spectral radiance used in this mask less MPLS platform, which utilizes a DMD slides projector (2500 ANSI lumens Optoma EP1690) as controlled light source set on the epi-fluorescence channel of a routine microscope (Zeiss Axiovert 40 CFL). The mixture behaves as a negative photoresist with higher absorption in the blue region of the visible spectrum.

SU-8(50)/S1818 was spin coated on a clean glass slide (Menzel-Glaser, Braunschweig, Germany) producing a processed film thickness of 200 μm. Two steps of soft baking at 65 °C for 5 min and 85 °C for 25 min, on a hotplate, were applied before sealing the walls of the micro chambers and inner structures to the glass substrate by exposing the film with a patterned source at 81 mJ·cm^−2^ through the glass slide. The samples were then flipped over and exposed from the photoresist side at 27 mJ·cm^−2^ to configure the monolithic roofs and ceilings. The samples were post-baked at 65 °C for 3 min and 85 °C for 15 min on a hotplate, developed by immersion in SU-8 developer (mr-dev 600, Microresist technology) for 2 h at room temperature and dried in N_2_ afterwards.

For the demonstration of color detection experiments, the chambers were filled with 0.1% solution of resazurine in water (Chroma-Gesellschaft, Münster, Germany), delivered with a 5 mL syringe set with a 0.5 mm diameter needle. While video imaged with the Nokia 6720 front camera, pH3 buffer solution (CertiPUR, Merck, Darmstadt, Germany) was delivered to the second reservoir. The acquired video stream was visually inspected to select few frames showing the progress of the color change.

### 2.3. Imaging

Three different cameras were used to test the adaptive lenses. The front and rear cameras of a Nokia 6720 classic cell phone and the frame embedded camera of a MacBook Pro Apple computer.

The front camera in the Nokia cell phone is a QVGA camera (320 × 240 pixels, 8 bit color channels for still images and 176 × 144 pixels in video mode) and it was operated with the software provided by the phone manufacturer, which produces .jpg pictures and .3gp format videos.

Nokia 6720's rear camera is a 5 MP, 2,592 × 1,944 pixels still camera with Carl Zeiss optics and autofocus for minimum focal distances of several centimeters. This camera is also capable of VGA video (640 × 480 pixels, 8 bits color channels) recording at 15 fps. The software provided with the phone has additional modes for this camera, which for still images was set in macro mode, maximum resolution and time-lapse acquisition at 6 fpm.

The MacBook Pro computer camera is a VGA device (640 × 480 pixels, 8 bits color channels) recording H.264 encoded videos in .mov format controlled by QuickTime Player Version 10.0 running on Mac OSX 10.6.7. The device was positioned at the center of the camera lens and the PDMS surface inherently adheres to the camera surface providing sufficient mechanical and optical coupling.

Liquid lenses were made of a drop of distilled water delivered with a 5 mL syringe (set with a 0.5 mm diameter stainless steel needle, Microlance 3 from BD Medial Systems, Drogheda, Ireland) directly on the PDMS reservoir.

All cameras lay horizontally and pointing upwards during the measurements, which proceeded immediately after creating the liquid lens. Measurements were carried out at 20 °C room temperature and normal indoors illumination of about 1,000 Lm/m^2^.

LOC samples were imaged in a routine inverted fluorescence microscope, Zeiss Axiovert 40 CFL (Carl Zeiss AG, Germany), with white light illumination and through a 10× (0.25) A-plan objective lens and a 2.5× objective. Images were acquired with a 5 MP, 8 bits color channels, Cannon PowerShot A95, mounted to the camera channel of the microscope.

Sessile drop evaporation was characterized by imaging the process with an Olympus SZ60 stereomicroscope at 6.3× magnification using an Au mirror on a 45° PDMS support. A distilled water drop was delivered on a PDMS surface and imaged at a 15 s interval with a Canon EOS 500D DSRL camera, acquiring 15 MP raw format images.

### 2.4. Data Processing

Acquired image sequences and video streams were manually inspected to determine best focus. Autofocusing code and plug-ins are available for automatic focus selection [23], however, for the purposes of this study the visual inspection of the captured image sequences was sufficient.

Image processing was carried out in 64-bit Image J 1.44014 and data collected and edited in Keynote 5.05 and Matlab 2008b.

Image composition was made by layer stacking in Photoshop CS4. 26 time-lapse images of the sessile drop evaporation were registered and manually masked with a 60% opacity brush to recover the drop surface reflections. Quantification of the drop chord and height was carried out in Image J for individual time-lapse acquisitions of the drop evaporation.

For the characterization of color reactions the images were collected in a single collage, split in color channels and the blue channel spatially averaged in Image J before profiling.

Modeling was carried out in Raytrace 2.21 (IME Software) for 2D ray tracing conceptual analysis and Atmos-Optical Design and Analysis Software [24] for lens design and characterization.

## 3. Results and Discussion

Adaptive optics is central in autofocusing cameras and can be implemented in different ways [14–17], but in all cases they are conceived as permanent components. In contrast, this work aims at a solution coherent with the evaluation of disposable LOC devices on intact cell phones. Accordingly, the adaptive optics concept we study in this work must be compatible with integration within the disposable LOC device.

Autofocus cameras in cell phones cannot focus at the short range required for LOC evaluation and an additional optical element should provide this function; however a permanent focusing element would introduce an extra component, which would restrict the ubiquity of the solution. Contrarily, the morphing lenses used in this work are versatile to adapt to different cell phone cameras, whereas they can be integrated in the LOC tests, and disposed together after use.

Figure 1(a) shows the schematics of the disposable morphing lens design. The LOC device is represented as mounted on a 1 mm thick substrate (e.g., standard glass slides) and separated from the lens-supporting element by a forward distance (*d_F_*) shorter than 2 mm, which defines a compact configuration for the entire arrangement. Ambient light is used for illumination.

The adaptive lens is created on a 150 μm thick PDMS layer that mildly adheres to the camera surface (Figure 1(b)) producing a reliable optical coupling between these elements. After the measurement the device is easily removed and disposed leaving the camera intact. The PDMS element is patterned with a 30 μm deep circular depression (800 μm diameter) that confines the lens to a fixed position (Figure 1(c)), and holds the LOC devices at a fixed distance from the camera.

The lens consists of a drop of distilled water delivered through a 0.5 mm needle. At its maximum volume in the operating regime the thickness of the lens along the optical axis is *h_1_* and for this condition the curvature is maximum, thus producing the shortest back focal length (*BFL_1_*).

Lens curvature is determined by the water drop volume and the contact angle with the substrate, which can be widely controlled using PDMS [25] as substrate. During natural evaporation the drop loses volume reducing its curvature and consequently increasing the BFL (*BFL_2_* in Figure 1(a)). During this process the BFL is scanned in a range sufficient to refocus the LOC device on diverse types of cameras.

At room temperature the 0.134 μL lens considered in Figure 1(a) takes several minutes to completely evaporate. This mechanism provides enough time for time-lapse image acquisitions or video recordings that capture different focusing conditions. In a post-processing step the best-focused images for each particular platform can be selected. Exactly the same procedure, and the same configuration, can be used on any cell phone or computer set, since the focal range that can be scanned with this design is large enough to accommodate optical differences across models and brands. In this work we corroborated this assumption on two different cameras of a standard cell phone (Nokia 6720 Classic) and the frame embedded camera of a MacBook Pro Apple computer.

**Figure 2.**
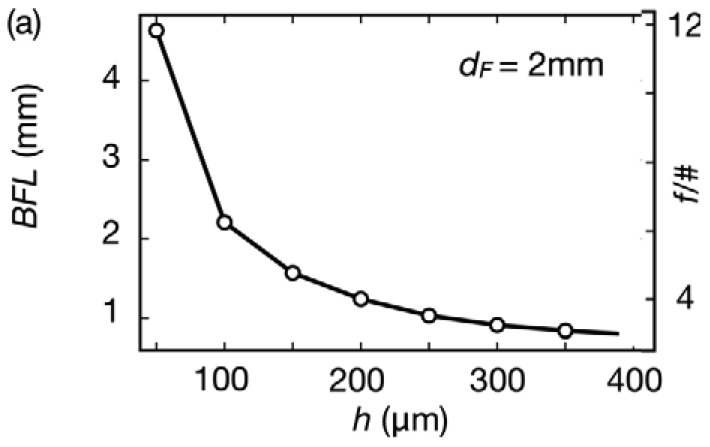

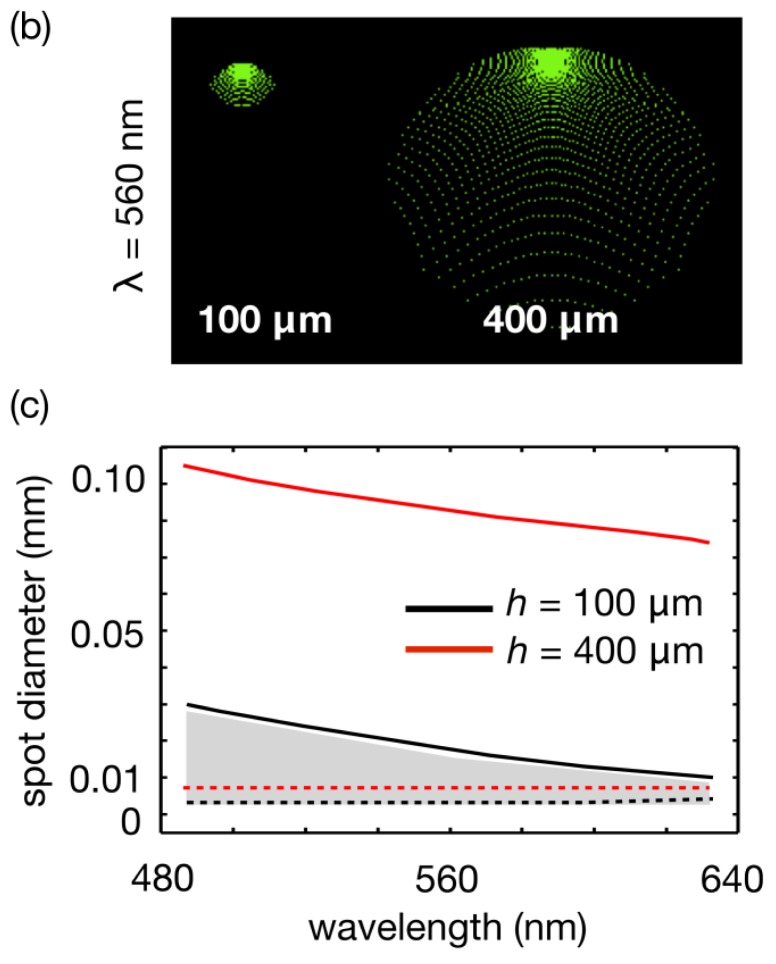
(**a**) Calculated back focal length (*BFL*) and aperture (f/#) *vs.* lens thickness along the optical axis (*h*); (**b**) Calculated spot diagram for λ = 560 nm and 2° angle for *h* = 100 μm and 400 μm; (**c**) RMS spot diameter in mm *vs.* wavelength for *h* = 100 μm in black solid lines and *h* = 400 μm in red solid lines. The diffraction-limited Airy diameter is indicated in dashed lines for the same two conditions.

Figure 2 collects the optical simulation of the lens shown in Figure 1(a). Figure 2(a) shows the behavior of the BFL for *h* values between 50 and 400 μm at a fixed *d_F_* of 2 mm. A small variation of 350 μm in *h* controls a BFL change of more than 4 mm. In the same way, the aperture of the system decreases with the curvature, implying a greater depth of field at longer BFLs (and smaller *h* values).

The spot diagram in Figure 2(b) characterizes the 2° distortions of the system for green light (*λ* = 560 nm), an intermediate range in the visible spectrum where these cameras operate. Not surprisingly, this simple configuration [26] suffers from multiple types of aberrations. The spot diagram in Figure 2(b) indicates spherical and coma aberrations increasing with the curvature (and thickness *h*), and the performance degrade towards shorter wavelengths.

The root mean square (RMS) spot diameter [27], the diameter of a circle containing approximately 68% of the focused energy, is shown in Figure 2(c), for the same curvatures as in Figure 2(b) and for different wavelengths. The dashed lines correspond to the diffraction-limited system (Airy diameters [28]), for the two previous curvatures.

As can be seen, the expected performance increases at longer wavelengths almost approaching the diffraction limit for 630 nm and *h* = 100 μm. Overall, the spot diameter is between 10 and 100 μm depending on the curvature and illuminating conditions, and under 30 μm for systems that operate with BFLs longer than 2 mm.

Airy diameters are between 4 and 8 μm for green light and above typical pixel sizes in standard cell phones, which can be between ∼1.23 μm width for 5 MP in 1/4 “format and ∼2 μm in 1/2.5”.

**Figure 3.**
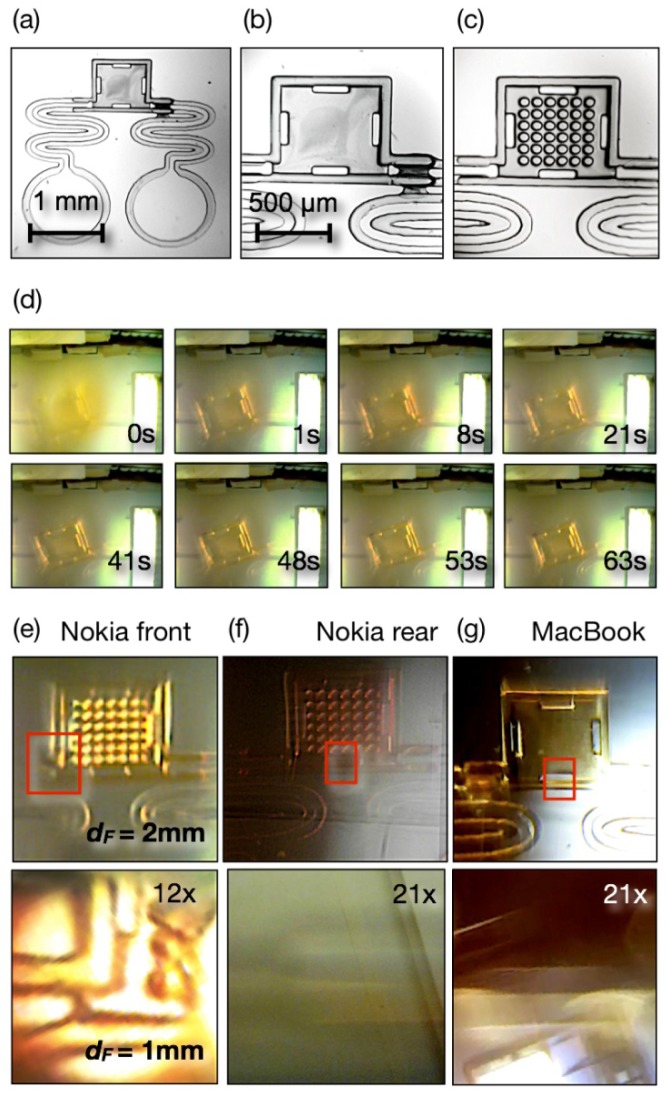
(**a**) Microscopy image of a 3D test microstructure at 25× magnification; (**b**) Detail at 100× magnification of one type of micro chamber used in this work; (**c**) Detail at 100× magnification of the second type of micro chamber used in this work; (**d**) Selected full frames (176 × 144 pixels, 8 bits per colour channel) from a 1 min video stream recorded with a Nokia 6720 cell phone front camera. The focus changes while the liquid lens evaporates and between 41 s and 53 s the best focus and depth of field is achieved; (**e**) Selected area of a microstructure imaged with a Nokia 6720 cell phone front camera operating in video mode for *d_F_* = 2 mm and *d_F_* = 1 mm in the lower panel, where an estimated 12× zoom factor is indicated; (**f**) Selected area of a microstructure imaged with a Nokia 6720 cell phone rear camera operating in 5 MP time-elapsed still image acquisition at 6 fpm for *d_F_* = 2 mm and *d_F_* = 1 mm in the lower panel, where an estimated 21× zoom factor is indicated; (**g**) Selected area of a microstructure imaged with a MacBook Pro camera operating in VGA (640 × 480 pixels) video mode for *d_F_* = 2 mm and *d_F_* = 1 mm in the lower panel, where an estimated 21× zoom factor is indicated.

The adaptive lens was experimentally tested imaging LOC microstructures of known dimensions. Figure 3(a) shows a 25× optical microscopy image of a SU-8/S1818 device fabricated with a refined 3D mask less micro prototyping MPLS method [22].

These structures have two parts: two open lateral flow service channels connected to 1 mm diameter reservoirs made of SU-8 (10) (30 μm thick) and a SU-8/S1818 3D chamber fused to the service channels. The chamber is 200 μm high and the whole system is a monolithic unit sealed to the glass slide substrate. The images were taken from the glass side.

Figure 3(b,c) are 100× magnifications of the chamber and display two different models tested in this work, an empty chamber (Figure 3(b)) and a chamber with a 6 × 6 array of 50 μm diameter pillars connecting the glass surface with the chamber ceiling.

Figure 3(d) shows selected full frames (174 × 144 pixels, 8 bits per channels color images) from a 1 min video stream acquired with the front camera of a standard Nokia 6720 cell phone. This video is acquired 2 min after the liquid lens was created and it was measured with a *d_F_* = 2 mm configuration. The video acquisition was controlled with the software provided with the phone.

The camera was focused in the background of the scene, which shows the ceiling of the room and a fluorescent lamp, which provided the illumination. No other source was employed in the experiments. Simultaneously, the area of the camera lens occupied by the liquid lens refocuses the test microstructure at different BFLs, while changing curvature due to evaporation. As can be seen, the sharpest images are obtained, with this camera, between 48 s and 63 s. It is also possible to observe that at the best focus the system maximized the depth of field, bringing together in focus the more than 100 μm high chamber ceiling and the 30 μm thick service channel, which became visible only after 48 s.

Depending on the location of the illumination, high contrast ratios exceeding the camera dynamic range, can occur in the region where the microstructure is imaged. Repositioning the cell phone is one possible supervised solution, whereas implementing high dynamic range (HDR) recording, which has been demonstrated for this same type of cell phone [12], could provide unsupervised imaging in future developments.

Figure 3(e–g) illustrate the performance of the three different cameras at imaging the microstructures using two different optical magnifications defined by the distance *d_F_* = 2 mm in the upper panels and *d_F_* = 1 mm, in the three lower panels. These images are the same selected areas cropped from full frames of different resolutions.

Figure 3(e) is taken from a 176 × 144 pixels frame video acquired with the Nokia 6720 front camera in white ambient light. Even at this low resolution, perhaps the most demanding condition one can propose, it was possible to resolve the 50 μm pillars with an acceptable depth of field. Shifting to *d_F_* = 1 mm provided a more than 12× optical zoom (red square in upper panel taken as reference) with enough depth of field to simultaneously capture the base and ceiling of the chamber. Note that the rectangular openings in the chamber were in the ceiling, more than 100 μm from the substrate surface where walls were imaged, and both could be identified.

Figure 3(f) is a still image taken from the rear 5 MP camera (2,592 × 1,944 pixels) of the Nokia 6720 cell phone. In order to find the right focus a time elapsed acquisition at 6 fpm was run. This acquisition mode is also available with the native software installed in the phone. In the *d_F_* = 2 mm range the edges were sharp, the 50 μm pillars could be better resolved and there was enough depth of field to capture channels and chambers in focus. One advantage of the 5 MP images is that they can tolerate digital zooming and image enhancement.

In the *d_F_* = 1 mm range a zoom factor of more than 21× could be estimated from the figure, although the depth of field was severely reduced in this condition, as illustrated by the focused image only at the edge of one the ceiling's rectangular openings.

Figure 3(g) shows the best-focused frames acquired with the MacBook Pro camera. In this case, a 640 × 480 pixels video was acquired at 15 fps with QuickTime Player, a free application from Apple.

The selected image for *d_F_* = 2 mm range showed an intermediate resolution between the two Nokia cameras, but with good depth of field and clarity. As in the case of the 5 MP Nokia camera the depth of field deteriorated at *d_F_* = 1 mm, and in this case the estimated zoom factor was also about 21×.

Sessile drop evaporation is a complex non-stationary phenomenon displaying more alternatives than the particular regime of operation considered so far. The drop geometry is defined by its contact angle with the host substrate, in this case a PDMS slab fabricated as detailed in the experimental section. As reported in [25] the contact angle with PDMS can be widely varied after UV irradiation if necessary to serve different design requirements.

During the evaporation of a liquid drop heat and mass transfer processes occur simultaneously, which involve associated heat transfer to the substrate, effect of the surface tension on convection within the drop, effect of contaminants and environmental conditions. Advanced modeling and detailed experiments on sessile drops are reported in the literature [18–20] and are beyond the scope of this work. Here, instead, drop evaporation was characterized for the conditions they have been used for optical detection.

**Figure 4.**
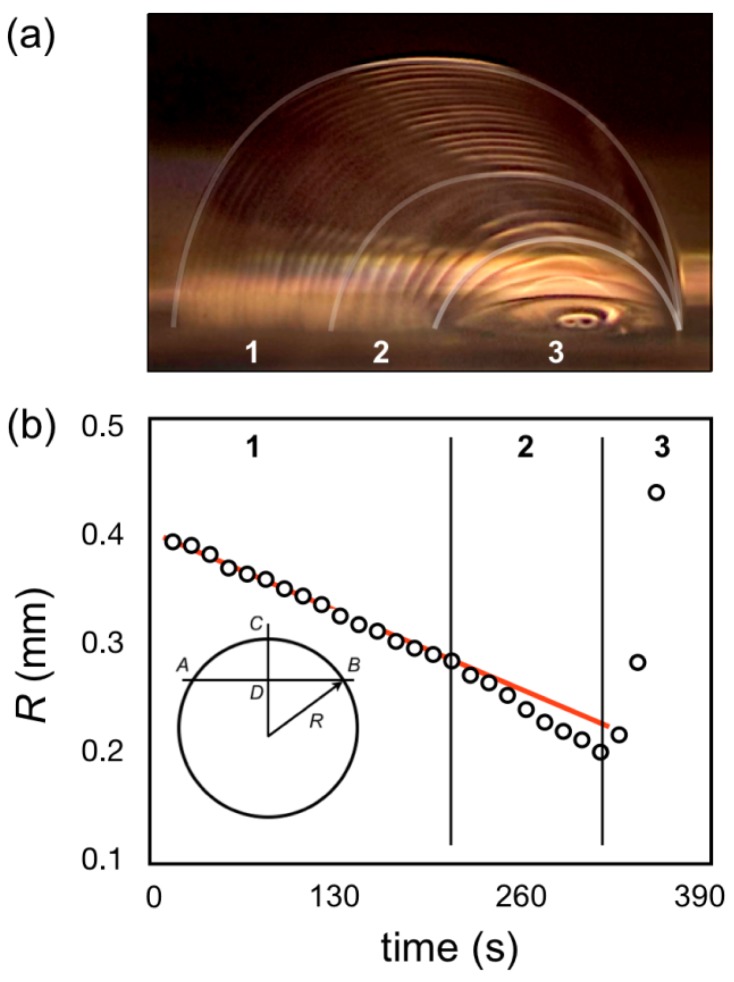
(**a**) Collection of sessile drop images captured with a stereomicroscope at a 15 s interval. The evaporation regimes (indicated as 1, 2 and 3) are highlighted; **(b)** Characterization of the drop evaporation by the drop radius of curvature, calculated from measured chord and height distances at 15 s intervals.

Figure 4(a) illsustrates the transition of a water drop on a PDMS substrate at room temperature as measured at 15s interval. The figure shows three different regimes in the behavior of the drop. In the first regime (1 in Figure 4(a)), occurring for about half of the recorded time, the contact angle remains practically constant and the evaporation changes the drop curvature by reducing the chord and height. In the second regime (2 in Figure 4(a)) the process accelerates, which can be noticed by the larger spacing between captures, and a noticeable change in contact angle, although the drop chord and height keep changing. Finally in the third regime (3 in Figure 4(a)) the changes accelerate even further, but the drop is pinned [18,19] at a constant chord and only the contact angle and associated height changes.

From measurements of chord and height taken from the individual pictures is possible to calculate the radius of curvature *R* as:

(1) $$R=\frac{{\left(\frac{d_{AB}}{2}\right)}^{2}+{\left(d_{CD}\right)}^{2}}{2\cdot d_{CD}}$$

where *d_AB_* is the chord and *d_CD_* is the height *h*. The evolution of *R* (Figure 4(b)) shows a linear decrease in the first regime (1 in Figure 4(b)), in what is characterized as an unpinned drop [19], where the contact line recedes, while the contact angle remains constant. In the second regime (2 in Figure 4(b)) the noticeable changes of contact angle in the still unpinned drop are reflected on a faster decrease of *R*. In the third regime (3 in Figure 4(b)) the drop is pinned and changes curvature at a faster rate producing a large range of increasing *R*s (and correlated BFLs), resulting in the conditions we exploit to adapt LOC imaging to diverse phone cameras.

Although the mechanism of de-pinning is beyond the scope of this work, the deviation from equilibrium of the contact angle is a correlated phenomenon [19]. On the other hand, the pinning drops are related to the surface roughness and eventual contamination, thus the pinning effect is expectable in most cases [19] and reliably present for the operating conditions of our devices.

Although our morphing lenses operate in the third time of regime, the results in Figure 4 suggests that other lens designs are possible, which could exploit slower focal changes to evaluate assays that demand longer response times.

Variations in ambient temperature and relative humidity can certainly affect the evaporation rate and regime of the focusing element. In the present conditions the systems operates satisfactory for normal air-conditioned environments, but if required the evaporation rate can be reduced by partially enclosing the lens environment.

In recent years a number of distributed microscopy principles associated with cell phones for imaging and communication have been demonstrated [6–8]. These examples employ dedicated instruments [7], reusable additional devices [6], operate with a single type of camera and require accurate sample positioning [13] or imply permanent modifications to the cell phones [8].

In contrast with these approaches the present principle is a disposable element integrated in the LOC device operating on intact cell phone cameras, and uses available ambient illumination as light source.

It is worth noticing that the performance of the adaptive lens is enough for the purpose it has been conceived: enables to image LOCs in the range of dimensions and resolutions that are required, it makes it adaptively and as part of a disposable device and operates in a compact and fixed configuration. The merit of the present solution is not the ultimate performance in each of these areas but the ability to collect all these aspects in a single disposable element, and establishing the compromises that make the *off-chip* LOC readout feasible on regular phones.

The capabilities of the conceived device to support sensing uses were tested for the detection of a transient chemical reaction within a LOC device.

To assess the limits of performance, the simpler front camera of the Nokia phone was used in video mode. The micro chamber in Figure 2(a,b) was imaged in this experiment at *d_F_* = 2 mm and 176 × 144 pixels color video resolution at 15 fps.

**Figure 5.**
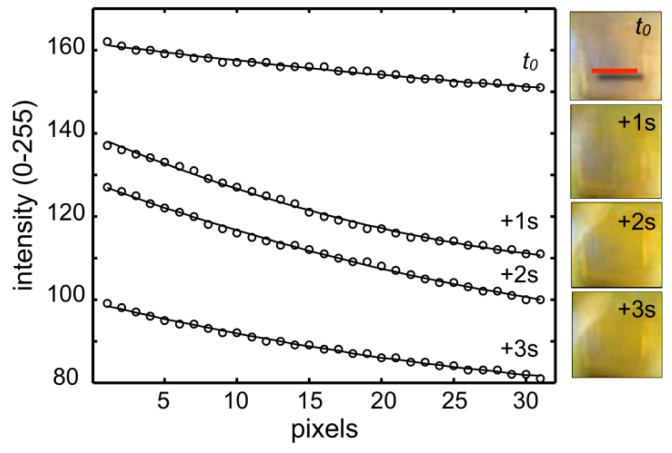
Color changing reaction of resazurine solution from pH7 to pH3 within a microchamber, captured at 15 fps with a Nokia 6720 front camera in video mode and analyzed through the blue camera channel.

A 0.1% solution of resazurine in water (pH 7, blue colour) was delivered to one reservoir of the micro chamber, after the adaptive lens was in the focusing region and the video acquisition had started. Once the solution reached the chamber and turned it blue, a pH3 buffer solution drop was delivered to the second reservoir and a colour change front (from blue to orange) crossed the measuring chamber. At 15 fps the reaction front could be captured in multiple frames, four of which are collected in Figure 5. These pictures show a blue chamber with a yellow front advancing from the right of the image, and completely crossing the chamber in about 3 s.

The color change can be more quantitatively rendered by profiling the blue channel of these images along a line (Figure 5, indicated in the *t_0_* panel). This result confirmed that a simple camera could follow a fast chemical reaction in the confined area (0.6 mm^2^) of a regular LOC element.

Summarizing, the results collected in Figure 3 show the ability of the considered concept to image micrometric features from 3D microstructures in the range representative of LOC detection areas, making the technique a feasible alternative for the evaluation of LOC devices on intact cell phones and computer sets without additional instrumentation or accessories, only using native software for acquisition and under available illumination.

These results demonstrate that a simple and generic device, integrating a morphing lens, can operate across diverse brands, models and types of compact cameras delivering the required performance for LOC sensing experiments.

The proposed concept is forgiving to imperfections, and delivers consistent results by complementing the versatile imaging and acquisition capabilities of intact cell phones. Further magnification is possible using thinner LOC substrates and approaching the specimen to the lens, such it has been demonstrated for fixed lenses with micro-positioned samples [13], however, the development in that direction certainly depends on the detection target, for general LOC evaluation the present range is sufficient and enables the use of robust substrates and classical LOC configurations, which are important aspects to enable ubiquitous LOC usage.

In this context, further progress would involve the incorporation of HDR acquisition to secure results in arbitrary illuminating conditions and the refinement of the fluidics to create the focusing element. Nevertheless, already at the present stage the possibility to detect transient chemical reactions within microstructures, using the simplest imaging configuration and the most common hardware and software resources readily available in cell phones, has been demonstrated.

## 4. Conclusions

Imaging of LOC micrometric features within regions larger than 500 × 500 μm^2^ has been demonstrated using ambient illumination and consumer cameras on intact cell phones and computer sets. The proposed method is generic and was conceived to adapt to diverse models and brands of pervasive consumer imagers using disposable devices that could be deployed in large numbers. The use of a morphing focusing element integrated in disposable LOC devices permitted their evaluation without introducing permanent accessories or specialized sample positioning, which would limit the ubiquity of cell phones as measuring platforms. The present concept highlights the possibility to materialize decentralized sensing relying on classical LOC technologies by temporarily co-opting intact cell phones as universal *off-chip* readers; In this case, tested with the monitoring of a transient chemical reaction using the simplest camera configuration.
